# Dietary habits, nutrient intake and biomarkers for folate, vitamin D, iodine and iron status among women of childbearing age in Sweden

**DOI:** 10.1080/03009734.2016.1201176

**Published:** 2016-07-07

**Authors:** Wulf Becker, Anna Karin Lindroos, Cecilia Nälsén, Eva Warensjö Lemming, Veronica Öhrvik

**Affiliations:** National Food Agency, P.O. Box, 75126 Uppsala, Sweden

**Keywords:** Diet, folate, iodine, iron, nutrition status, vitamin D, women

## Abstract

**Background:**

Dietary intake and nutritional status are important for pregnancy and pregnancy outcomes. Dietary advice on folate, targeted to women of childbearing age, aims at preventing neural tube defects in the offspring.

**Aim:**

To describe food and nutrient intake and nutritional status among women of childbearing age in Sweden in relation to current nutrition recommendations.

**Methods:**

Dietary intake was assessed using a web-based four-day consecutive food record among adults aged 18–80 years—‘Riksmaten 2010–11 adults’. In a subsample, biomarkers of folate, vitamin D, iodine, and iron status were assessed.

**Results:**

Women of childbearing age had lower intakes of fruit and vegetables, fish, and whole grains, but higher intakes of soft drinks. Macronutrient composition was generally in line with the Nordic Nutrition Recommendations, except for a lower intake of fibre, a higher intake of saturated fatty acids, and added sugars. Mean intakes of vitamin D, folate, and iron were below recommended intakes (RI). Median urinary iodine concentration (UIC) was 74 μg/L, 20% had insufficient vitamin D status, and 3% low folate concentrations with no age differences. Furthermore, 29% of women 18–44 years of age had depleted iron stores.

**Conclusions:**

The dietary pattern among women of childbearing age (18–44 years) was less favourable compared to older women. Intakes of some micronutrients were below RI, but no differences in vitamin D, folate, or iodine status between age groups were observed. However, improvements of folate and iodine status among women of childbearing age are warranted. This can be achieved by following dietary guidelines including use of folic acid-containing supplements.

## Introduction

Dietary intake and nutritional status among women affect pregnancy and pregnancy outcomes. Dietary guidelines in Sweden targeted to women of childbearing age include advice on folic acid supplementation (1). Adequate folate status is important for prevention of neural tube defects (NTD) in the offspring (2). For pregnant women recommended intakes of several micronutrients are increased (3), and sufficient status prior to conception is therefore also important. This is the case for e.g. iodine and iron, for which there are valid biomarkers. Iodine and iron deficiencies are still major nutritional problems world-wide (4,5). About 30% of the world’s population is estimated to have insufficient iodine intake, with increased risk of adverse effects on growth and mental development. The prevalence of iron deficiency (without anaemia) varies between 5% and 17% in industrialized countries, and young women are especially at risk. Iron deficiency precedes anaemia. Anaemia is associated with fatigue, reduced mood and cognitive function, and poor pregnancy outcomes and quality of life (5).

In the recent nation-wide dietary survey ‘Riksmaten 2010–11 adults’ biomarkers of these nutrients were collected for the first time. In this paper, data on food consumption, nutrient intake, and status for vitamin D, folate, iodine, and iron among Swedish women are presented with a focus on the childbearing age (18–44 years).

## Materials and methods

### Subjects

In the Riksmaten adults 2010–11 survey, a representative sample of 5,000 individuals aged 18–80 years and living in Sweden were invited to participate. The data collection took place between May 2010 and July 2011. Food consumption was captured using a web-based food record during four consecutive days (6). A total of 1797 subjects (36%) completed the food record, 1005 women (41%) and 792 men (31%). A subgroup of 1008 individuals, constituting one-fifth of the total sample, were invited to take part in a biomonitoring project. Statistics Sweden (SCB) divided the population sample into seven regions according to affiliation to Swedish Occupational and Environmental Medicine Centres (OEMCs). Each region included the regional capital (Linköping, Lund, Stockholm, Umeå, Uppsala, Gothenburg, and Örebro) together with two randomly selected counties in each region. An equal number of individuals was selected in each region independently of population size. Recruitment took place at four different occasions during the year to cover different seasons. Twelve individuals per region and occasion were invited to participate. Out of the 1008 individuals that were invited (7 regions, 3 places in each (the capital and 2 counties), 4 occasions, and 12 individuals per round), a total of 300 subjects (30%) agreed to participate, with a higher participation rate (33%) among women. See Table 1 for details of number of women in different age groups.

**Table 1.** Number of women completing the food record and for whom analytical data on biomarkers are available.

Age group	Food record	P-folate	Ery-folate	P-25(OH)D	UIC	P-ferritin
18–30 years	203	28	26	27	26	28
31–44 years	247	38	35	37	37	38
45–64 years	357	54	53	51	50	54
65–80 years	198	30	27	29	27	30
All	1005	150	141	144	140	150

### Dietary assessment

The participants reported everything they ate and drank for four consecutive days, using a web-based food diary developed by the National Food Agency (NFA). Food intake and status in all seasons and all days of the week were captured by carrying out the study from May 2010 to July 2011 and by randomly assigning starting days to participants. The level of education was somewhat higher among participants than among non-participants, and drop-out was also higher among e.g. subjects born outside Sweden. More details on demographic characteristics have been given by Amcoff et al. (6).

### Nutritional biomarkers

*Folate*. Whole-blood samples for erythrocyte folate status were collected in EDTA tubes and plasma folate samples in PST tubes by nurses at the OEMCs. Erythrocyte folate concentrations were analysed at the Karolinska University Hospital, Sweden, using an immunoassay kit (Roche Folate III, Roche Diagnostics GmbH, Mannheim, Germany). Plasma folate was analysed at Uppsala University Hospital, Sweden, using a chemiluminescence immunoassay method on an Abbott Architect ci8200 analyser with reference standard (cat. no. 1P74-35, Abbott Laboratories, Abbott Park, IL, USA).

*Vitamin D*. Plasma 25-hydroxy vitamin D (P-25(OH)D) was analysed at Vitas, Oslo, Norway with LC-MS (7). The assay is accredited by the Vitamin D External Quality Assessment Scheme. P-25(OH)D was calculated as 25(OH)D_2_ + 25(OH)D_3_.

*Iodine*. Urinary iodine concentration (UIC) was determined in duplicate by the same laboratory technician at the Sahlgrenska Academy, Department of Internal Medicine and Clinical Nutrition, Gothenburg, Sweden. The method we used was a modified Sandell–Kolthoff method (8), where iodide is used as a catalyst in the reduction of ceric ammonium sulphate (yellow colour) to the cerous form (colourless) in the presence of arsenious acid. The rate of colour disappearance is directly proportional to iodide concentration.

*Ferritin.* Iron status was measured as the plasma ferritin concentration using a chemiluminescent microparticle immunoassay (CMIA) (Architect^®^, Abbott Laboratories) at Uppsala University Hospital, Uppsala, Sweden.

### Assessment of nutrition status

*Folate*. An erythrocyte folate concentration <317 nmol/L and a plasma folate concentration <6.8 nmol/L are considered to indicate insufficient status (3).

*Vitamin D*. Plasma 25(OH)D concentrations <50 nmol/L) are considered to indicate insufficient vitamin D status (3).

*Iodine*. According to the World Health Organization (WHO), a median UIC of 100–200 μg/L indicates sufficient iodine status in a population (9).

*Iron*. Ferritin concentrations <12 μg/L in children and adolescents and <15 μg/L in adults are indicative of depleted iron stores (3,10) in the absence of inflammation.

### Statistical analyses

All statistical analyses were done in STATA (version 11.2 and 12). Shapiro–Wilks tests were used to determine normality. Non-parametric tests Kruskal–Wallis and Wilcoxon rank–sum were used, and ANOVA and *t* test were used for normally distributed variables.

## Results

There were minor, and non-significant, differences between subjects in the subsample and other participants with regard to weight, height, and BMI.

### Intake of foods and nutrients

**Figure 1.**
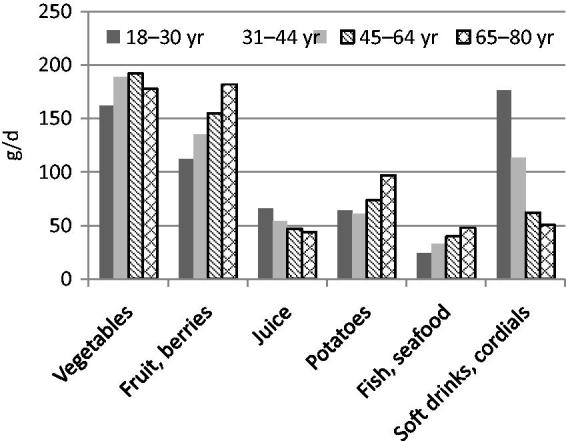
Mean intake (g/d) of selected foods among women in Riksmaten 2010–11.

**Figure 2.**
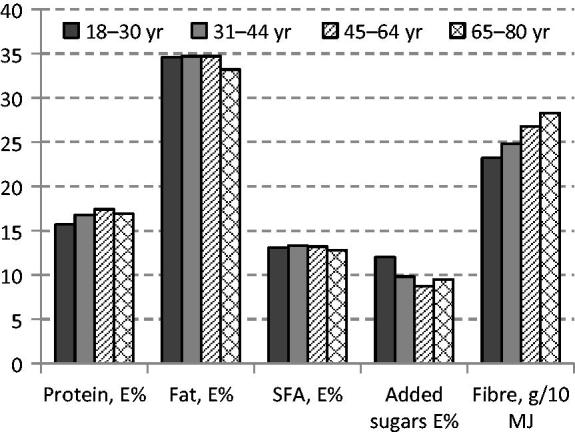
Intake of protein, total fat, saturated fatty acids (SFA), added sugars (as E%), and dietary fibre (g/10 MJ) among women in Riksmaten 2010–11.

The intake of fruit and vegetables, potatoes, fish, and seafood was lower, while intake of juice, soft drinks, and cordials was higher among women of childbearing age (18–44 years) than among older women (45–80 years) (Figure 1). Reported energy intake tended to be higher in the younger age groups. The proportion of macronutrients was relatively similar across age groups. However, intake of added sugars was higher, and intake of dietary fibre and whole grains was lower in the younger age groups (Figure 2; Table 2).

**Table 2.** Intake of energy, added sugar, whole grains, vitamin D, folate, iron, and heme-iron among women in Riksmaten 2010–11. Mean per day and per 10 MJ.

		Energy	Whole grains		Vitamin D		Folate		Iron		Heme-iron	
Age group	*n*	MJ/d	g/d	g/10 MJ	μg/d^a^	μg/10 MJ	μg/d^a^	μg/10 MJ	mg/d	mg/10 MJ	mg/d	mg/10 MJ
18–30 years	202	7.6	35	45	5.2	6.7	223	298	8.9	11.9	0.99	1.38
31–44 years	247	7.6	38	52	6.2	8.3	247	334	9.7	12.9	1.21	1.62
45–64 years	358	7.3	40	56	6.6	9.2	263	365	9.9	13.8	1.19	1.62
65–80 years	198	7.1	43	60	7.6	10.7	275	388	9.4	13.3	1.12	1.62
All	1005	7.4	39	54	6.4	8.8	253	349	9.5	13.1	1.14	1.57

a Excluding supplements.

Reported daily energy intake among women in the subsample was higher than among women not providing blood samples (7.8 versus 7.4 MJ; *P =* 0.038). There were no differences in gross dietary composition, e.g. per cent energy from protein, fat, carbohydrate, and alcohol, and dietary fibre (g/10 MJ) and sodium (mg/10 MJ).

### Folate intake and status

**Figure 3.**
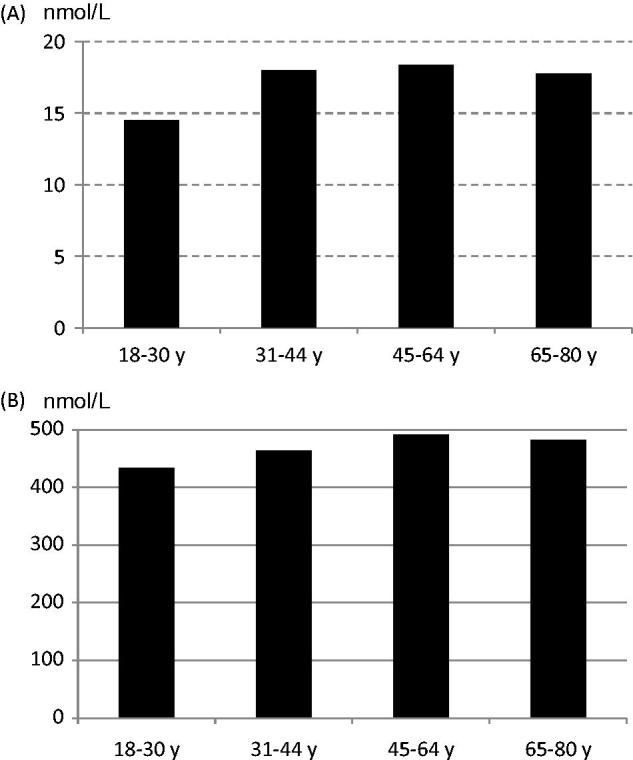
Folate status among women in the subsample. A: mean plasma folate; B: mean erythrocyte folate concentration.

Women of childbearing age (18–44 years) had a lower folate intake than women aged 45–80 years (*P <* 0.001) (Table 2). Increasing intake of fruit and vegetables was associated with a higher folate intake. The mean erythrocyte folate concentration was 470 nmol/L (SD 117) (*n =* 141), and mean plasma folate concentration was 17.5 nmol/L (SD 11.0) (*n* = 150). Low blood folate concentrations (erythrocyte-folate <317 nmol/L; plasma-folate <6.8 nmol/L) were found in 3% of the women. Despite significantly different folate intakes there were no differences in folate status between women aged 18–44 and 45–80 years (Figure 3). However, women reporting a fruit and vegetable intake above 500 g/d had a higher folate status (*R^2^* = 0.26, *P =* 0.002).

### Vitamin D intake and status

**Figure 4.**
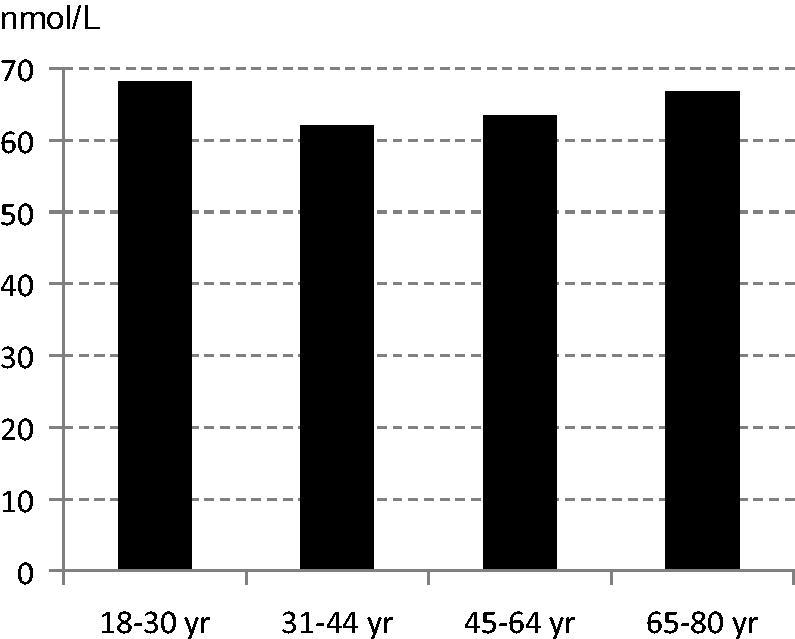
Means of plasma 25(OH)D (nmol/L) among women in the subsample.

The reported mean intake of vitamin D from the diet among women was 6.4 μg/d lower among the younger age groups (*P <* 0.001) (Table 2). However, there were no differences in vitamin D status, assessed with plasma 25(OH)D (Figure 4). Insufficient vitamin D status (plasma 25(OH)D <50 nmol/L) was found in 20%.

### Iodine status

In the subsample of participants in the Riksmaten 2010–11 study the median UIC among women (*n =* 140) was 74 μg/L. Altogether 66% had a UIC below 100 μg/L. Mean creatinine-adjusted UIC was 102 μg/g. No intakes were calculated due to incomplete values for iodine contents in foods in the NFA food composition database.

### Iron status

Intakes of total and heme-iron (mg per day and mg per 10 MJ and day) were similar among women of childbearing age (18–44 years) and older women (Table 2), and intakes did not differ in the subsample. However, there was a difference between these age groups in mean plasma ferritin concentrations, 41 μg/L (SD 34) and 92 μg/L (SD 74), respectively (*P <* 0.001). Among women of childbearing age, 20% had ferritin values ≤12 μg/L and 29% <15 μg/L, indicative of depleted iron stores.

## Discussion

The low participation rate (41%) in the food consumption survey and in the biomarker subsample (33%) may have influenced the results of this survey. However, all age groups were well represented. The level of education was somewhat higher among participants than among non-participants. The reported intake of fruit, berries, vegetables, and whole grains was generally lower compared to dietary guidelines, while the intake of sugary and fatty foods with a high content of saturated fat and salt was high. Women of childbearing age reported lower intakes of fruit and vegetables, fish, and whole grains, and higher intake of soft drinks compared to older women.

### Folate

Reported mean folate intake was generally above the average requirement (200 μg/d), but below recommended intake (300 μg/d), especially for women of childbearing age (400 μg/d). Even adjusting for apparent under-reported intakes, few subjects reached the last-mentioned intake. Sixteen women of childbearing age reported intake of folic acid supplements (4%) regularly or sometimes, and 117 reported intake of multivitamin supplements regularly or sometimes. Among women in the subsample two (3%) reported intake of folic acid supplements and 16 intake of multivitamins. According to data from the Swedish birth register, about 15% of women reported use of folic acid supplements during early pregnancy in 2012, compared to about 1% in 1999 (11). In a study of 1098 pregnant women in the south-west of Sweden, carried out in 2013, 30% reported that they used folate-containing supplements during the first trimester (12). In an interview study carried out in early 2010 comprising 1000 adults, drawn from a representative sample of Swedish households, 36% of the women reported taking supplements, of which 32% contained folic acid (NFA, unpublished). Thus, about one-tenth took folic acid-containing supplements. The study also showed that the knowledge of when a woman should start taking folic acid prior to the planning of pregnancy was low.

In Sweden, the prevalence of children born with NTD has decreased since the 1970s. Since 1999, abortions are included in the statistics, and there has been a decrease up to 2013 (11). This is in contrast to data for many European countries, where no clear trend has been observed (13). Data on folate status in the Riksmaten subsample, however, indicate that many women of childbearing age would benefit from improved status in order further to reduce the risk of NTD. This can be achieved by adhering to the general dietary guidelines and also using folic acid-containing supplements and choosing fortified products. Results from calculations based on weekly menus complying with dietary guidelines and including choice of keyhole-labelled food products (14) suggest that such a diet would result in a folate intake of about 400 μg/d for an adult woman (unpublished). However, this represents an ideal food pattern, which implies substantial changes in the typical diet of today.

### Vitamin D

Mean vitamin D intake was below recommended intake in all age groups, lowest among the youngest women (18–30 years). Despite this, there were no age differences in plasma 25(OH)D, and levels indicated a sufficient status (≥50 nmol/L) for the majority. Under-reporting could be one explanation, also more frequent outdoor activities and thereby contribution from sun exposure.

### Iodine

The median UIC of 74 μg/L in the Riksmaten subsample is below the WHO range of 100–200 μg/L and would thus indicate an insufficient status (9). The lower limit of 100 μg/L is based on an average urine volume of 1.5 L/d, which then corresponds to an intake of about 150 μg/d, equal to the recommended intake in the Nordic Nutrition Recommendations (NNR) 2012 (3). However, UIC is dependent on osmolality, and the daily fluid intake may vary considerably. Using a urine volume of 1.5 L the observed UIC of 74 μg/L would correspond to 111 μg/24 h, which is above the average requirement (100 μg/d). Alternatively, using data for the creatinine-adjusted UIC (102 μg/g) and mean 24-h creatinine excretion reported for German adults (15), the median 24-h iodine excretion has been estimated to be around 130 μg. This is still below the recommended intake (3). A decreasing trend in iodine content in Swedish milk has been reported, which is in line with data from Swedish Market Basket studies, indicating a decrease in the average iodine supply from food and beverages (16). Thus, a general increase in iodine intake is desirable, especially important for women of childbearing age. Use of iodized salt in home cooking and food products by manufacturers and increased fish consumption are feasible options. Also, efforts to maintain and possibly restore iodine levels in milk and dairy products are warranted.

### Iron

In the Riksmaten subsample there was a non-significant tendency for lower dietary intake of total iron and heme-iron in the youngest age group (18–30 years) compared to women aged 45–80 years. Plasma ferritin levels were significantly lower among women of childbearing age, and the proportion with ferritin concentrations <12 μg/L and <15 μg/L was 20% and 29%, respectively. In Sweden sifted flour was fortified with carbonyl-iron until the beginning of 1995. Sjöberg and Hulthén (17) compared food habits, iron intake, and status in two cross-sectional studies among 15–16-year-old girls and boys, sampled from schools in Gothenburg, before (1994, *n =* 1245) and after (2000, *n =* 1020) discontinuation of the fortification. Among girls the reported intake of both total and heme-iron was lower in the year 2000. Prevalence of iron deficiency, defined as serum ferritin <15 μg/L, was higher (45%) in 2000 than in 1994 (37%). There were, however, no data on mean serum ferritin levels. Reported levels of total and heme-iron intake are, however, lower compared to the Riksmaten sample. In a longitudinal study on Swedish adolescents (60 males and 66 females), carried out from 1993 to 1999, changes in iron status were measured from 15 to 21 years of age. In females, median serum ferritin increased significantly, after a non-significant decrease at 17 years, from 27 μg/L at 15 years to 34 μg/L at 21 years (18). In females, prevalence of iron deficiency, defined as serum ferritin <12 μg/L, was 18%, 26%, and 21% at 15, 17, and 21 years, respectively. In the Riksmaten subsample the median serum ferritin level among women aged 18–30 years was 36.5 μg/L. Thus, it was similar to the value for the 21-year-old women in the study by Samuelson et al. (18). Taken together, the results from these studies indicate that young women would benefit from a diet with a higher iron density and availability.

## Conclusions

Young adults are an important target group for public health work, since food habits are established early in life and women of that age might bear children. In the Riksmaten 2010–11 study, women of childbearing age reported less favourable food habits and lower intakes of some micronutrients compared to older women. No or only minor differences in vitamin D, folate, and iodine status between age groups were observed. However, iron status was lower among the younger age groups. The present data indicate that folate, iron, and iodine status among women of childbearing age needs improvement. This can be achieved by following dietary guidelines including use of folic acid-containing supplements.
